# Draft Genome Sequence of *Streptococcus pyogenes* Strain 06BA18369, a Human Pathogen Associated with Skin and Soft Tissue Infections in Northern Canada

**DOI:** 10.1128/genomeA.00387-13

**Published:** 2013-06-27

**Authors:** Ryan R. McDonald, George R. Golding, James Irvine, Morag R. Graham, Shaun Tyler, Michael R. Mulvey, Paul N. Levett

**Affiliations:** Saskatchewan Disease Control Laboratory, Regina, Saskatchewan, Canadaa; Department of Biology, University of Regina, Regina, Saskatchewan, Canadab; National Microbiology Laboratory, Winnipeg, Manitoba, Canadac; Department of Medical Microbiology, University of Manitoba, Winnipeg, Manitoba, Canadad; Population Health Unit, Mamawetan Churchill River & Keewatin-Yatthé Health Regions & Athabasca Health Authority, LaRonge, Saskatchewan, Canadae; Department of Academic Family Medicine, University of Saskatchewan, Saskatoon, Saskatchewan, Canadaf

## Abstract

We report the draft sequence of *Streptococcus pyogenes* 06BA18369 (*emm* type 41.2, sequence type 579 [ST579]), isolated from a skin and soft tissue infection (SSTI) mixed with *Staphylococcus aureus*. This genome provides insight into the genetic composition of *S. pyogenes* strains associated with mixed SSTIs.

## GENOME ANNOUNCEMENT

*Streptococcus pyogenes* can cause a variety of infections in humans that range from mild superficial infections to severe invasive disease. *S. pyogenes* isolates are uniformly susceptible to penicillin but have demonstrated resistance to other classes of antimicrobials, including macrolides, tetracyclines, and lincosamides (1–3). Epidemiological surveillance of *S. pyogenes* in Canada has identified *emm* type 1 as its leading cause of invasive infections (4). Recently, an epidemic of invasive *emm* type 59 infections has been described in Canada (4, 5). Concurrently, mixed skin and soft tissue infections (SSTI) associated with *S. pyogenes emm* type 41.2 and *Staphylococcus aureus spa* type t311 were found to be prevalent in a region of northern Canada that is endemic for community-associated methicillin-resistant *S. aureus* (CA-MRSA) (6, 7).

In this study, one mixed SSTI case was selected for further analysis, and *S. pyogenes* 06BA18369 (*emm* type 41.2; sequence type 579 [ST579]) was selected for genome sequencing. Whole-genome shotgun sequencing was performed with the 454 Life Sciences GS-FLX genome sequencer (Roche Applied Science, Laval, Quebec, Canada). The initial read assembly by Newbler v1.1.03.24 (Roche) generated 59 contigs of >500 bp. Gap closure was initiated by Sanger sequencing DNA inserts of clones isolated from a fosmid genomic DNA library. Sequence reads spanning the contig gaps were assembled using Gap4, provided in the Staden package (8). The final draft genome sequence consists of 47 contigs that includes a combined 1,813,404 bases with 38.4% G+C content. Contig sequences were analyzed for the presence of putative protein-coding sequences, tRNA genes, and rRNA regions with GeneMark (9), tRNAscan (10), and RNAmmer (11), respectively. GeneMark identified 1,840 putative coding sequences, while 30 tRNA genes and three rRNA regions were recognized.

Annotation of contig sequences indicated the presence of the *emm*-like genes *mrp* and *enn*. Phylogenetic analysis and genetic organization of the three *emm*-like gene sequences were consistent with the *emm* pattern D group, which includes strains associated with skin and soft tissue infections (12). Contig sequences were queried for prophage content using PHAST (13), revealing the presence of five regions consistent with a known prophage. These prophages are similar to those identified in other sequenced genomes with respect to integrase gene homology and virulence factor content. Four putative prophages harbor integrase-encoding genes, each showing 100% sequence identity with phage-1, phage-2, phage-3, and prophage-like element SpyCIM53 of *S. pyogenes* strain Alab49 (14), and carry the virulence factor genes *speL*, *speM*, *speC*, *mf2*, and *mf3*. The fifth prophage is similar to *S. pyogenes* strain MGAS315 phage 315.6 (15), based upon *int* sequence identity (100%) and the presence of the virulence factor gene *sdn*.

*S. pyogenes* 06BA18369 also carries a *cas*-CRISPR array that spans the gap between two contigs. As such, this array consists of at least seven spacers, all but one being homologous to known prophage sequences detected in either other *S. pyogenes* strains or *Streptococcus agalactiae* strains.

This draft genome sequence adds to the collection of genomes of *S. pyogenes* strains that originate from skin and soft tissue infections.

### Nucleotide sequence accession numbers. 

This Whole-Genome Shotgun project has been deposited at GenBank under the accession no. APMZ00000000. The version described in this paper is the first version, accession no. APMZ01000000.

## References

[1] Torres RS Torres RP Smeesters PR Palmeiro JK de Messias-Reason IJ Dalla-Costa LM 2011 Group A *Streptococcus* antibiotic resistance in southern Brazil: a 17-year surveillance study. Microb. Drug Resist. 17:313–319 21417774 10.1089/mdr.2010.0162

[2] Brenciani A Bacciaglia A Vecchi M Vitali LA Varaldo PE Giovanetti E 2007 Genetic elements carrying *erm*(B) in *Streptococcus pyogenes* and association with *tet*(M) tetracycline resistance gene. Antimicrob. Agents Chemother. 51:1209–1216 17261630 10.1128/AAC.01484-06 PMC1855496

[3] Pérez-Trallero E Montes M Orden B Tamayo E García-Arenzana JM Marimón JM 2007 Phenotypic and genotypic characterization of *Streptococcus pyogenes* isolates displaying the MLSB phenotype of macrolide resistance in Spain, 1999 to 2005. Antimicrob. Agents Chemother. 51:1228–1233 17242142 10.1128/AAC.01054-06 PMC1855467

[4] Tyrrell GJ Lovgren M Forwick B Hoe NP Musser JM Talbot JA 2002 M types of group A streptococcal isolates submitted to the National Centre for Streptococcus (Canada) from 1993 to 1999. J. Clin. Microbiol. 40:4466–4471 12454137 10.1128/JCM.40.12.4466-4471.2002 PMC154642

[5] Tyrrell GJ Lovgren M St Jean T Hoang L Patrick DM Horsman G Van Caeseele P Sieswerda LE McGeer A Laurence RA Bourgault AM Low DE 2010 Epidemic of group A *Streptococcus* M/*emm*59 causing invasive disease in Canada. Clin. Infect. Dis. 51:1290–1297 21034198 10.1086/657068

[6] Golding GR Levett PN McDonald RR Irvine J Quinn B Nsungu M Woods S Khan M Ofner-Agostini M Mulvey MR, Northern Antibiotic Resistance Partnership 2011 High rates of *Staphylococcus aureus* USA400 infection, northern Canada. Emerg. Infect. Dis. 17:722–725 21470471 10.3201/eid1704.100482 PMC3377391

[7] McDonald RR Golding GR Levett PN Yost CK Irvine J Horsman GB Mulvey MR, the Northern Antibiotic Resistance Partnership 2008 Characterization of *Streptococcus pyogenes* and *Staphylococcus aureus* isolates associated with mixed skin and soft tissue infections in northern Saskatchewan. Can. J. Infect. Dis. Med. Microbiol. 19:77–142

[8] Bonfield JK Smith KF Staden R 1995 A new DNA sequence assembly program. Nucleic Acids Res. 23:4992–4999 8559656 10.1093/nar/23.24.4992 PMC307504

[9] Besemer J Borodovsky M 2005 GeneMark: web software for gene finding in prokaryotes, eukaryotes and viruses. Nucleic Acids Res. 33:W451–W454 15980510 10.1093/nar/gki487 PMC1160247

[10] Schattner P Brooks AN Lowe TM 2005 The tRNAscan-SE, snoscan and snoGPS web servers for the detection of tRNAs and snoRNAs. Nucleic Acids Res. 33:W686–W689 15980563 10.1093/nar/gki366 PMC1160127

[11] Lagesen K Hallin P Rødland EA Staerfeldt HH Rognes T Ussery DW 2007 RNAmmer: consistent and rapid annotation of ribosomal RNA genes. Nucleic Acids Res. 35:3100–3108 17452365 10.1093/nar/gkm160 PMC1888812

[12] Bessen DE Sotir CM Readdy TL Hollingshead SK 1996 Genetic correlates of throat and skin isolates of group A streptococci. J. Infect. Dis. 173:896–900 8603968 10.1093/infdis/173.4.896

[13] Zhou Y Liang Y Lynch KH Dennis JJ Wishart DS 2011 PHAST: a fast phage search tool. Nucleic Acids Res. 39:W347–W352 21672955 10.1093/nar/gkr485 PMC3125810

[14] Bessen DE Kumar N Hall GS Riley DR Luo F Lizano S Ford CN McShan WM Nguyen SV Dunning Hotopp JC Tettelin H 2011 Whole-genome association study on tissue tropism phenotypes in group A *Streptococcus*. J. Bacteriol. 193:6651–6663 21949075 10.1128/JB.05263-11 PMC3232895

[15] Beres SB Musser JM 2007 Contribution of exogenous genetic elements to the group A *Streptococcus* metagenome. PLoS One 2:e800 17726530 10.1371/journal.pone.0000800 PMC1949102

